# Bioavailability, pharmacokinetics and safety of riociguat given as an oral suspension or crushed tablet with and without food

**DOI:** 10.1186/2050-6511-16-S1-A82

**Published:** 2015-09-02

**Authors:** Soundos Saleh, Reiner Frey, Corina Becker, Sigrun Unger, Georg Wensing, Wolfgang Mück

**Affiliations:** 1 Clinical Pharmacology, Bayer Pharma AG, 42113 Wuppertal, Germany; 2 Global Biostatistics, Bayer Pharma AG, 42113 Wuppertal, Germany

## Background

Riociguat is the first oral, soluble guanylate cyclase stimulator licensed for the treatment of pulmonary arterial hypertension (PAH) and chronic thromboembolic pulmonary hypertension [1-3]. For some patients with PAH, e.g. children and the elderly, swallowing tablets may be inappropriate or difficult; therefore, oral suspension and crushed tablet formulations of riociguat were developed. Here we present data from two single-centre, randomised, non-blinded, crossover studies evaluating the relative bioavailability of riociguat as oral liquid and standard “immediate release” (IR) oral tablet under fed and fasted conditions, and as a crushed-tablet preparation versus oral tablet under fasted conditions.

## Methods

In Study 1, 30 healthy male and female volunteers received five single doses of riociguat in a randomised order: 0.3 and 2.4 mg riociguat (0.15 mg/mL suspension), 0.15 mg riociguat (0.03 mg/mL suspension), and 1.0 mg riociguat IR tablet (all in a fasted state), and 2.4 mg riociguat (0.15 mg/mL suspension) after a high-calorie breakfast. In Study 2, 25 healthy male volunteers received four single doses of riociguat 2.5 mg in a randomised order: oral IR tablet with water and crushed tablet suspended in applesauce or crushed tablet suspended in water (all fasted), and oral whole IR tablet after a continental breakfast. Repeated blood samples for pharmacokinetic assessment were taken. Both studies also assessed safety and tolerability.

## Results

In Study 1, dose-normalised pharmacokinetic parameters of riociguat suspensions were almost identical to the standard 1.0 mg IR tablet in fasted conditions (Table 1); 90% confidence intervals for the ratio ‘suspension/IR tablet’ area under concentration (AUC) versus time curve and maximum drug concentration in plasma (C_max_) of riociguat were within the bioequivalence reference range (80–125%). After food intake, dose-normalised AUC and C _max_decreased by 15% and 38%, respectively. In Study 2, riociguat exposure of the 2.5 mg IR tablet was similar for all four modes of administration (Table 2); AUC ratios ‘crushed tablet suspended in water or applesauce/IR tablet’ were within bioequivalence criteria. C_max_ was increased by 17% after administration of crushed tablet suspended in water. Food intake decreased C_max_ of riociguat IR tablet by 16% with unaltered AUC versus the fasted state. The most frequently reported drug-related adverse events (AEs) were related to riociguat mode of action, notably headache (Study 1: 30–47%; Study 2: 13–20%). There were no deaths, serious AEs or withdrawals owing to AEs.

**Table 1:** Study 1: Pharmacokinetic parameters of riociguat in plasma after a single oral dose of riociguat (pharmacokinetic analysis population, n=29):

Parameter	Unit	n	2.4 mg high-concentration suspension fasted	n	2.4 mg high-concentration suspension fed	n	0.3 mg high-concentration suspension fasted	n	0.15 mg low-concentration suspension fasted	n	mgIR tablet fasted
AUC	μ*h/L	29	781/49 (230–1515)	29	663/45 (258–1212)	27	91/43 (38–165)	25	47/60 (8.3–91)	29	311/46 (103–587)

AUC/D	h/L	29	0.33/49 (0.10–0.63)	29	0.28/45 (0.12–0.50)	27	0.30/43 (0.13–0.55)	25	0.31/60 (0.056–0.61)	29	0.31/46 (0.10–0.59)

C_max_	μ/L	29	78/31 (43–128)	29	48/22 (32–70)	29	9.8/33 (4.5–17)	29	4.9/35 (1.9–8.1)	29	36/30 (18–61)

C_max_/D	1/L	29	0.03/31 (0.02–0.05)	29	0.02/22 (0.01–0.29)	29	0.03/33 (0.01–0.06)	29	0.03/35 (0.01–0.05)	29	0.04/30 (0.02–0.06)

t_max_^a^	h	29	1.5 (0.75–4.0)	29	4.0 (3.0–12)	29	1.0 (0.50–3.0)	29	1.0 (0.50–4.0)	29	1.0 (0.50–3.0)

t_1/2_	h	29	9.2/41 (2.8–21)	29	9.4/33 (4.2–17)	27	7.8/39 (3.5–15)	25	6.8/42 (2.5–12)	29	7.9/43 (3.4–14)

Data presented as geometric mean/% coefficient of variation (range) unless otherwise specified.

^a^ Median (range)

AUC, area under the concentration versus time curve; AUC/D; AUC divided by dose; C_max_, maximum drug concentration in plasma; C_max_/D: C_max_ divided by dose; IR, immediate-release; t_max_; time to reach Cmax; t_1/2_; half life

**Table 2:** Study 2: AUC and C_max_ ratios of ‘crushed tablet suspended in water or applesauce/whole IR tablet’ (fasted) and ‘whole IR tablet fed/whole IR tablet fasted’ after a single oral dose of riociguat (pharmacokinetic analysis population, n=24):

Ratio	Parameter	Unit	CV	Estimated ratio (%)	90% CI
Crushed tablet suspended in applesauce/whole tablet fasted	AUC	μ*h/L	16.76	98	(91–107)
	C_max_	μ/L	18.77	88	(80– 96)
Crushed tablet suspended in water/whole tablet fasted	AUC	μ*h/L	16.76	103	(95–112)
	C_max_	μ/L	18.77	117	(107–128)
Whole tablet fed (continental breakfast)/whole tablet fasted	AUC	μ*h/L	16.76	96	(89–104)
	C_max_	μ/L	18.77	84	(77–92)

AUC, area under the concentration versus time curve; C_max_, maximum drug concentration in plasma

CI, confidence interval; CV, coefficient of variation; IR, immediate release

## Conclusions

Riociguat bioavailability was similar between the high- (0.15 mg/mL) and low- (0.03 mg/mL) concentration suspensions and the IR tablet. Riociguat exposure was also similar between whole IR tablet and the crushed tablet suspended either in water or applesauce. Minor food effects were observed for the high-concentration (2.4 mg) suspension and the 2.5 mg IR tablet, but overall the pharmacokinetic results suggest that riociguat formulations are interchangeable.
